# Del Nido Cardioplegia Versus Cold Blood Cardioplegia in Adult Cardiac Surgery: Protocol for a Randomized Controlled Trial

**DOI:** 10.2196/17826

**Published:** 2020-07-14

**Authors:** Jessica Garcia-Suarez, Javier Garcia Fernandez, Sergio Sanz, Daniel Martinez Lopez, Leticia Reques, Alberto Forteza Gil

**Affiliations:** 1 Department of Anesthesiology and Critical Care Puerta de Hierro Majadahonda University Hospital Madrid Spain; 2 Department of Cardiovascular Surgery Puerta de Hierro Majadahonda University Hospital Majadahonda, Madrid Spain

**Keywords:** del Nido cardioplegia, cardiac surgery, myocardial protection

## Abstract

**Background:**

The use of cardioplegia solutions as a myocardial protection technique is essential during cardiac surgery with cardiopulmonary bypass. The del Nido cardioplegia solution (DNS) has been widely used as a myocardial preservation technique for pediatric patients undergoing cardiac surgery with cardiopulmonary bypass. Its unique pharmacological features have created growing interest for adult cardiac surgery, especially for elderly patients or those with ventricular dysfunction who are more prone to ischemia-reperfusion injury. Ever since its implementation, several retrospective studies have been published to validate the efficacy, safety, and efficiency of DNS in adult patients undergoing coronary revascularization, valve replacement, or combined procedures. Recently, a meta-analysis based on nine retrospective studies was published claiming the noninferiority of DNS compared to other conventional cardioplegia solutions. Few prospective randomized studies have been conducted whose primary outcome was the assessment of DNS clinical efficacy compared to other solutions commonly used in adult patients.

**Objective:**

The aim of this randomized clinical trial is to assess the benefits of DNS compared to Cardi-Braun blood cardioplegia solution in clinical and biochemical terms regarding myocardial protection during adult cardiac surgery.

**Methods:**

This is the protocol of a controlled, randomized, single-center clinical trial carried out at the Puerta de Hierro Majadahonda University Hospital in Spain. A total of 474 participants over the age of 18 years undergoing elective cardiac surgery with cardiopulmonary bypass will be assigned to groups by simple randomization to receive either DNS or Cardi-Braun blood cardioplegia solution. The primary outcome will be the differences between groups in myocardial protection in biochemical terms (ie, perioperative troponin levels) and clinical terms (ie, presence of the composite variable *acute cardiovascular event*). The clinical trial will be carried out under conditions of respect for the fundamental rights of the person and the ethical principles that affect biomedical research with human beings, as well as in accordance with international recommendations contained in the Declaration of Helsinki and its subsequent revisions.

**Results:**

The inclusion process started in 2018. Data cleaning and analyses are expected to take place in the fall of 2020 and the results are expected in January 2021.

**Conclusions:**

This study is particularly relevant as it will be one of the first to analyze the clinical effects of del Nido cardioplegia on the basis of direct myocardial protection parameters. In light of published studies, carrying out prospective studies based on primary clinical objectives with a larger sample, high-risk patients, and longer cardiopulmonary bypass times continues to be necessary. We believe that our study addresses an important gap in the knowledge of del Nido cardioplegia in adult patient cardiac surgery and will be able to clarify the possible benefits of this method in a large population of patients undergoing these procedures.

**Trial Registration:**

European Union Drug Regulating Authorities Clinical Trials Database (EudraCT) 2017-005144-14; https://www.clinicaltrialsregister.eu/ctr-search/search?query=2017-005144-14+; ClinicalTrials.gov NCT04094168; https://clinicaltrials.gov/ct2/show/NCT04094168

**International Registered Report Identifier (IRRID):**

DERR1-10.2196/17826

## Introduction

### Scientific Background and Explanation of Rationale

Myocardial protection during cardiac surgery with cardiopulmonary bypass is essential to avoid detrimental effects of ischemia secondary to aortic cross-clamping. To do so, several strategies have been used throughout the years, such as intermittent aortic occlusion, induced ventricular fibrillation, deep systemic hypothermia, or the administration or cardioplegia solutions.

In 1955, Melrose et al were the first to propose the concept of hyperkalemic cardioplegia, using a high-concentration potassium-citrate solution to afford elective reversible cardiac arrest [1]. Ever since, multiple pharmacological solutions and modes of administration have been studied, without currently having a clear and homogenous model considered a reference standard.

On the other hand, there has been a tangible change in the features of patients undergoing cardiac surgery for the last two decades. Patients are older and, therefore, have increased comorbidity, and they undergo more complex surgical procedures, which entail longer ischemia times. The quest for a cardioplegia solution providing maximal myocardial protection has never been more important.

The del Nido cardioplegia solution (DNS) was first formulated for pediatric cardiac surgery in the early 1990s and has been widely used ever since [2]. It contains the crystalloid Plasmalyte, with an electrolyte composition similar to that of the extracellular space. Its mannitol content reduces myocardial cell inflammation and serves as an ischemia induced-free-radical scavenger. Magnesium sulfate improves ventricular recovery by preventing excessive intracellular calcium to build up by blocking calcium channels. The presence of lidocaine, which blocks sodium channels, prolongs the myocyte refractory period. The formula provides adequate myocardial protection for ischemia periods of up to 90 minutes.

Its pharmacological properties have contributed to its popularity for elderly patients with depleted myocardial reserve undergoing cardiac surgery [3,4]. Experimental studies carried out in aged rabbit hearts showed further accumulation of calcium and, therefore, an undermined ventricular function after prolonged ischemic periods. DNS used with the same experimental population showed decreased intracellular calcium levels and fewer spontaneous ventricular contractions [5].

Several retrospective studies have assessed the safety and efficacy of DNS in adults undergoing coronary revascularization, valve replacement, combined procedures, and cardiac reinterventions [6-8].

In 2017, a meta-analysis comparing the effects of DNS and conventional cardioplegia solutions in adult cardiac surgery was published [9]. It concluded that there were shorter aortic cross-clamp times, reduced cardiopulmonary bypass times, reduced need for mechanical ventilation, and shorter intensive care unit (ICU) lengths of stay for the DNS group. On the other hand, no significant differences were observed as far as the following were concerned: the evolution of cardiac enzymes, postoperative vasoactive needs, the appearance of atrial fibrillation, in-hospital lengths of stay, or mortality. DNS appeared to be noninferior in terms of myocardial protection, morbidity, and mortality. Nevertheless, the results are somewhat restricted due to the lack of randomized prospective studies in the analysis.

Recently, two prospective studies assessing the effects of DNS in adults have been published [10,11], yet their primary outcomes focused on myocardial protection indirectly or on biochemical data (ie, electrical activity, need for ventricular defibrillation after the release of the aortic cross-clamp, and troponin evolution after surgery). Light has yet to be shed on the clinical advantages of DNS in adult patients, including older patients and those with a history of ventricular dysfunction.

### Aim of the Study

The aim of this study is to establish the benefits of DNS in clinical and biochemical terms regarding myocardial protection during adult cardiac surgery.

## Methods

### Study Setting

The study was planned according to the updated Consolidated Standards of Reporting Trials (CONSORT) statement, the Declaration of Helsinki, and the Guidelines of Good Clinical Practice issued, as required, by Spanish regulatory authorities. This study will be conducted at one center, the Puerta de Hierro Majadahonda University Hospital.

### Trial Design

**Figure 1:**
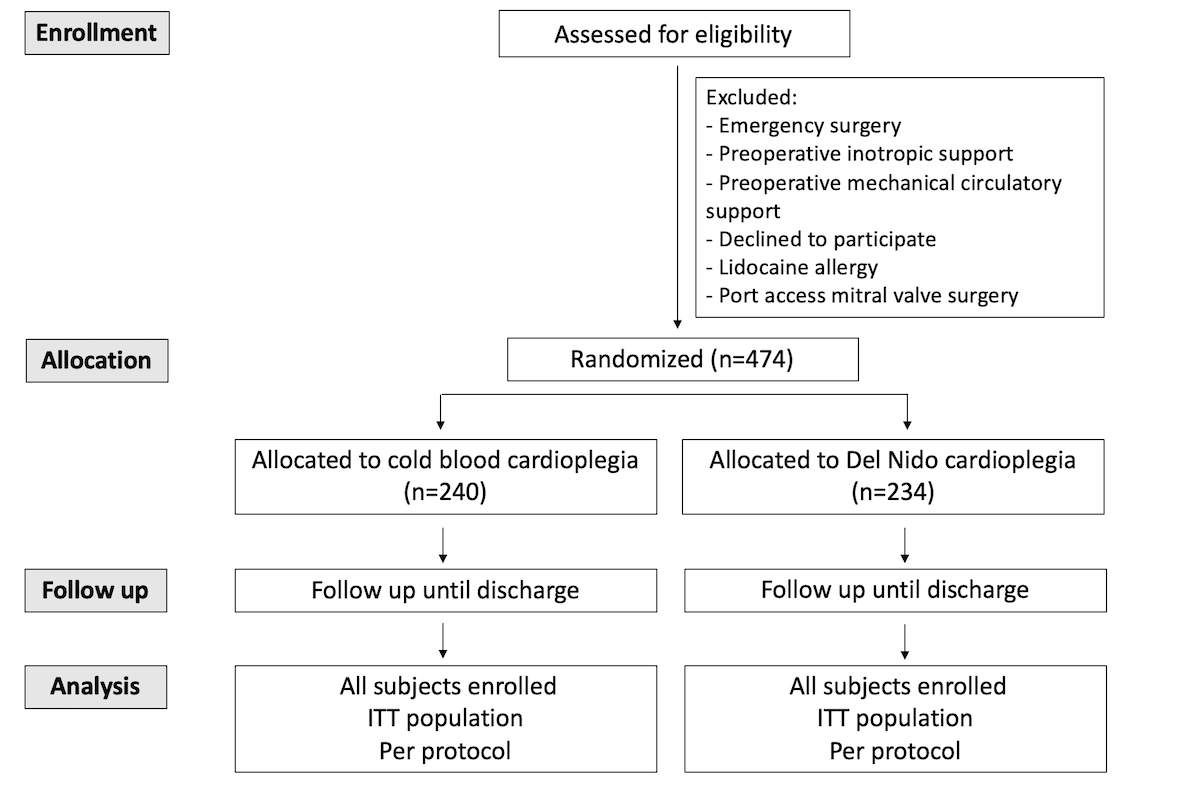
CONSORT flow diagram.

This study is designed as a single-center, double-blind, controlled clinical trial. Participants will be randomized into a group receiving del Nido cardioplegia (intervention group) or a group receiving Cardi-Braun cold blood cardioplegia (control group; Figure 1). We will conduct a superiority analysis.

### Eligibility Criteria for Participants

All patients over the age of 18 years who are scheduled for cardiac surgery may be eligible to participate in the study. The inclusion and exclusion criteria are explained in detail in Textbox 1 below.

**Inclusion and exclusion criteria for study participants.**

**Inclusion criteria**

- Patients over the age of 18 years
- Patients undergoing elective cardiac surgery with cardiopulmonary bypass

**Exclusion criteria**

- Patients who do not consent to participate in the study
- Patients with a documented allergy to amide-type local anesthetics
- Patients undergoing emergent surgery
- Unstable patients who require pharmacological inotropic support; mechanical circulatory support, such as intra-aortic balloon counterpulsation (IABC) or extracorporeal membrane oxygenation (ECMO); or preoperative intubation
- Patients undergoing mitral valve replacement surgery using the Heartport technique

### Study Procedures and Interventions

Within the control group, blood cardioplegia will be administered by means of a cold induction dose at 4-8 °C and a maintenance dose administered every 20 minutes. It will be dispensed via the anterograde or retrograde approach according to the protocol of the center.

Within the intervention group, del Nido cardioplegia will be administered according to the usual practice in a single dose of 1000 cc via the anterograde or retrograde approach according to the protocol of the center. Additional doses (550 mL) will be administered when spontaneous electrical activity appears or, in the case of periods of ischemia, longer than 90 minutes. Common clinical practice will not be modified in the remaining perioperative aspects.

### Outcome Measures

The main variables of the study will be the composite clinical variable *acute cardiovascular event* and the biochemical variable *maximum postoperative troponin value*.

The *acute cardiovascular event* variable will consist of one or several of the following events:

1. Early perioperative myocardial infarction: occurring during the first 72 hours after surgery and defined according to the criteria of the Task Force of the European Society of Cardiology, the American Heart Association, and the World Heart Federation.
2. Prolonged low cardiac output: defined as the need to maintain support with two or more inotropic and/or other circulatory mechanical assistance devices—intra-aortic balloon counterpulsation (IABC) or extracorporeal membrane oxygenation (ECMO)—beyond the first 24 postoperative hours.
3. Appearance of ventricular arrhythmias: ventricular tachycardia (VT) and ventricular fibrillation (VF) requiring electrical cardioversion in the first 24 postoperative hours.

To analyze the effect of cardioplegia on myocardial protection at a biochemical level, the level of troponins will be monitored upon arrival at the ICU and during the first 48 hours. Likewise, the maximum troponin peak will be collected during the first 48 postoperative hours.

Other secondary variables of efficacy and safety will be analyzed. Among the intraoperative variables, surgical times (ie, ischemia time and cardiopulmonary bypass times), the volume of cardioplegia solution administered and its route of administration, temperature, average blood pressure, and maximum blood glucose values, as well as the need for electrical cardioversion after aortic cross-clamp release, will be monitored.

Among the postoperative variables, the occurrence of atrial fibrillation will be registered, as well as the requirement for inotropic and mechanical pharmacological support, time to withdrawal, and the occurrence of clinical complications (ie, deterioration of ventricular function upon hospital discharge, surgical reintervention, prolonged mechanical ventilation, acute kidney injury, infectious complications, convulsive activity, or stroke). The need for ICU readmission, ICU and hospital length of stay, and mortality will also be noted.

Subgroup analyses will be carried out in four populations: elderly patients (over 80 years), patients undergoing prolonged cardiopulmonary bypass (longer than 120 minutes), patients with low ventricular ejection fraction (less than 30%), and patients subjected to coronary artery bypass grafting.

### Participant Timeline

Follow-up will be carried out from the beginning of the randomization until the participant is discharged. The variables will be collected in the operating room postoperatively; variable collection will occur at a higher frequency within the first 48 hours.

The research team will monitor the possible adverse events, including the time of occurrence, duration, intensity, course, and outcome, in order to make an assessment of the causal relationship between the adverse event and the drug.

### Statistical Analyses

#### Sample Size Calculation

The literature reviewed reflects a need for postoperative inotropic vasoactive support ranging from 35% to 40%. In the sample size calculation, we propose that better myocardial protection will be reflected in a lower need for electrical cardioversion, lower pharmacological inotropic or mechanical support, and a lower incidence of myocardial infarction.

For a hypothetical reduction of inotropic support within the first 24 postoperative hours from 38% to 25%, with an α risk of 5%, at a statistical power of 80% (β=.20), by bilateral test with application of the Fleiss correction factor, and estimating losses of 10%, 474 participants distributed into two groups will be necessary.

Participants will be assigned to groups by simple randomization of the sample, obtaining a computer sequence by Stata 16 (StataCorp). The computer distribution will assign 234 participants to the intervention group (DNS) and 240 participants to the control group (blood cardioplegia). When patients arrive at the theater, perfusionists will enroll participants.

#### Analyses

A description of the data from each group of the study and the total sample will be carried out. The properties of the original variables will be defined, and the aforementioned composite variables will be created. The main variable—*prolonged low cardiac output syndrome*—will be created from the following original variables: *presence of two or more inotropic agents at 24 hours of admission*, *mechanical support with IABC*, and/or *mechanical support with ECMO or other ventricular assistance*. The main composite variable—*acute cardiovascular event*—will be created from the following variables: *perioperative myocardial infarction*, *prolonged low cardiac output syndrome*, *prolonged vasoplegia*, and/or *VT or VF episodes in the first 24 hours*.

The quantitative variables shall be described through the mean (SD), median (IQR), 95% CI, and minimum and maximum values. Qualitative variables shall be presented as relative and absolute frequencies. To ensure the uniformity of the groups, a detailed analysis will be made by evaluating the association using the Pearson correlation coefficient and the *P* value by means of the Student-Fisher *t* test; correction for unequal variances for the quantitative variables, and the association with the odds ratio and the *P* value, will be obtained with chi-square tests for the categorical variables.

The raw effect of each type of cardioplegia on the acute cardiovascular event will be estimated using a logistic regression model.

Two populations will be defined through intention-to-treat analysis and by biological efficacy. The first population will include all the initially randomized subjects, including all those who leave the study due to noncompliance. The second population will include all those subjects who complete the study within the same group in which they were randomized. An intermediate safety analysis will be performed when approximately 50% of patients have been randomized.

### Monitoring and Quality Assurance

The clinical trial is defined as a *low intervention–level trial*, due to the fact that the cardioplegia solutions used are already approved and used in accordance with the terms of the marketing authorization; as well, the solutions are extensively backed up by published scientific evidence on their safety and efficacy (Royal Decree 1090/2015).

The confidentiality of the identity of the volunteers will be respected if the data obtained in this study are published. Subjects’ data included in the study will be treated in accordance with Organic Law 15/1999, December 13, on the Protection of Personal Data.

### Ethics Approval and Consent to Participate

This study has been registered in the European Union Drug Regulating Authorities Clinical Trials Database (EudraCT) (No. 2017-005144-14) and at ClinicalTrials.gov (No. NCT04094168). The study protocol has been approved by the Ethics Committee of Puerta de Hierro Majadahonda University Hospital and was approved by the Spanish Medicines and Health Products Agency (AEMPS).

This clinical trial will be carried out under conditions of respect for the fundamental rights of the person and the ethical principles that affect biomedical research with human beings, as well as in accordance with international recommendations contained in the Declaration of Helsinki and its subsequent revisions. Likewise, national recommendations will be followed in accordance with the guidelines of the AEMPS.

Participants will receive information, both orally and in writing, regarding the manner in which the study will be carried out, the study aims, possible risks that may arise from the study, and the rules that must be observed during the follow-up period. If the patient fulfils the inclusion criteria and agrees to participate in the study, she or he will provide written informed consent; providing consent will not prevent the patient from withdrawing such consent and abandoning the study at any time and for any reason.

### Availability of Data and Materials

The datasets used and/or analyzed during the study will be available from the corresponding author upon reasonable request.

## Results

The inclusion process started in 2018. Data cleaning and analyses are expected to take place in the fall of 2020 and the results are expected in January 2021.

## Discussion

### Overview

This study is expected to expand knowledge of DNS in adult cardiac surgery. DNS is assumed to be beneficial in terms of myocardial protection, and it is expected to corroborate a reduction in VF after declamping the aorta and in the number of defibrillations. Also, it is presumed to result in a reduction in postoperative troponin levels and inotropic support for low cardiac output syndrome. Although it is not the main objective of the study, the trial will provide information regarding the benefits of DNS in high-risk subpopulations, such as elderly patients and those with longer periods of cross-clamping or low ventricular ejection fraction.

This study is particularly relevant as it will be the first to analyze the clinical effects of del Nido cardioplegia on the basis of direct myocardial protection parameters. Although the meta-analysis published in 2017 highlighted some clinical benefits in favor of del Nido cardioplegia, there was a fundamental limitation, which is that data collection was based on retrospective studies.

Further published prospective studies also failed to focus their primary objectives on the analysis of the possible clinical effects of del Nido cardioplegia. Ad et al analyzed the postoperative troponin peak and the need for electrical cardioversion after aortic cross-clamp release in a population of 89 patients; in addition to its small sample size, this study excluded high-risk patients over 80 years of age, and the data showed short aortic clamp times [10].

Sanetra et al analyzed troponin peak and electrical activity after aortic cross-clamp release in a population of 150 patients undergoing aortic valve replacement. All clinical outcomes detailed in the study were part of the secondary objectives. Likewise, they excluded from their analysis reoperated patients with ejection fractions under 30% or with coronary artery disease susceptible to percutaneous interventionism or surgery [11].

In light of the above studies, carrying out prospective studies based on primary clinical objectives with a larger sample, high-risk patients, and longer cardiopulmonary bypass times continues to be necessary [12]. We believe that our study addresses an important gap in the knowledge of del Nido cardioplegia in adult patient cardiac surgery and will be able to clarify the possible benefits of this method in a large population of patients undergoing these procedures.

### Strengths and Limitations of This Study

This clinical trial has several strengths. The calculated sample size is large and includes elderly patients with a history of ventricular dysfunction. It does not exclude complex procedures that may involve longer cardiopulmonary bypass times.

The trial also has some limitations. Although surgical procedures will be performed by different surgeons, the single-center design may generate external validation problems. The exclusion of emergent interventions and of patients with preoperative vasoactive support also limits extrapolation of results to unstable patients.

In developing the methodology, we have aimed to reduce bias as much as possible. The compilation of other myocardial protection variables has been fundamental in addressing bias.

## Abbreviations

AEMPS: Spanish Medicines and Health Products Agency
CONSORT: Consolidated Standards of Reporting Trials
DNS: del Nido cardioplegia solution
ECMO: extracorporeal membrane oxygenation
EudraCT: European Union Drug Regulating Authorities Clinical Trials Database
IABC: intra-aortic balloon counterpulsation
ICU: intensive care unit
VF: ventricular fibrillation
VT: ventricular tachycardia
